# **Complex Governance Does Increase Both the Real and Perceived Registration Burden: The Case of the Netherlands** Comment on "Perceived Burden Due to Registrations for Quality Monitoring and Improvement in Hospitals: A Mixed Methods Study"

**DOI:** 10.34172/ijhpm.2020.264

**Published:** 2021-01-06

**Authors:** Patrick PT Jeurissen, Niek Klazinga, Luc Hagenaars

**Affiliations:** ^1^Radboud Institute of Health Sciences (RIHS), Nijmegen, The Netherlands.; ^2^Amsterdam Medical Center, Amsterdam, The Netherlands.

**Keywords:** Quality Registrations, Administrative Burden, Hospitals, Managed Competition, Netherlands

## Abstract

The burden of registrations for professionals should be more firmly on the policy agenda. In a rigorous study, Marieke Zegers and colleagues make a compelling argument why that should be the case. In Dutch hospitals, the average professional spends 52.3 minutes a day on quality registries and monitoring instruments. Many more administrative duties exist. These represent substantial resources and ultimately could become a drag on the intrinsic motivation of the care professions. We agree with Zegers et al that we are in need for more operational efficiency. However, the issue at hand is very complex and also intensely connected to the entire healthcare system and its different levels. More operational efficiency alone will not solve this problem. We are also in need for better governance of data-issues at the macro-system level.

## Introduction

 Marieke Zegers and colleagues made an excellent contribution to the expanding literature of the (rising) administrative burden in healthcare. They present an empirical study on the burden of quality monitoring by healthcare professionals. Results are sobering. They find a substantial time burden of data handling for nurses and physicians (52.3 minutes a day); only 25% of such quality measures are primarily registered for quality improvement; 36% of the measures were perceived as useful for improving quality in everyday practice; and, 57% of all quality registrations are primarily used for accountability purposes. They also find that perceived unreasonable registrations negatively correlate with joy in work and with more distraction from actual time for treating patients, although and as a possible bias less intrinsically motivated professionals might hold more negative feelings on quality registrations.^1^ Nevertheless, the net performative forces of these registrations might actually be negative.^2^ The methodological rigor of the study presents few reasons to doubt the accuracy of the measurements.

 As policy implications, Zegers et al do plea for: (1) less quality registrations, (2) a more limited set of core indicators, and (3) a better use of information and communication technologies to reduce these workloads. Thus, they propose for a higher level of operational efficiency in quality data collections, for example to be achieved by additional investments in administrative support for the registration process. However, in recent decades the number of quality and safety registrations has only increased. We think that the wickedness of this problem asks for more to be done. Besides more operational efficiency, we argue that adequate governance of data and information is of the utmost importance to tackle the root causes of this problem. The complex interactions between the different levels within the healthcare system and the lack of routine statistics on the total costs of registration on all levels, might enhance the administrative burdens to unreasonable levels or the other way around. The more so because this administrative burden lacks an explicit ‘price’ and is buried within the official cost statistics. The importance of this governance issue also comes to the fore as a result of large diversity in numbers of data custodians, purchasers and oversight agencies and their responsibilities. In other words, we feel that administrative burdens on the clinical level may not only reflect operational inefficiencies, but also failures in governance at the macro- (and meso) level.

###  Adequate Information Might Improve the Public Good

 Over the past decades, the amount of data has increased tremendously. Due to the rapid expansion of information and communications technology technologies, the costs of processing and analyzing data has been reduced tremendously. Partly as a result, data requests have gone up. However, although it has become more convenient to process and analyze data, this was not (necessarily) the case for those that had to deliver and fill for the increasing number of data requests. The more so since the number of data-hungry stakeholders also increased due to reforms that articulated the purchasing functions and due to the coming of new players such as oversight bodies, patient associations, accreditation organizations, and data companies.

 In principle, adequate information might contribute to solve important agency problems, including those that relate to the quality and safety of our healthcare. Adequate information also holds potential to help increase the allocative efficiency of scarce resources, for example through active purchasing of high-value care.^3,4^ However, and as stated above, the gathering of information is not for free and comes with a ‘price.’

###  Total Indirect and Administrative Costs Are Very High

 At the macro-level, multiple purchaser models typically bear higher administrative expenses than single purchaser systems, because of economies of scale and scope. Other reasons lay in higher costs for billing and claims (that are often absent or limited in single purchaser systems), and in confidentiality practices when purchasers and providers compete with each other. The administrative burden bared on the level of financers and regulators is around 4% in the Netherlands. This is much lower than in other multiple-payer systems such as Germany and especially the United States.^5^

 However, such statistics do only partly resemble the total administrative burden. At the meso-level, providers do also employ many staff without responsibilities for patient treatments. In the United States – where administrative expenses have proven to be an important determinant for the excessive costs of the total healthcare system^6^ – in one study only hospital administrative costs add up to 25% of turnover; and the Netherlands does not come out that well either, with almost 20% they are second in line.^7^ In theory such comparatively high administrative costs at the provider level might partly be compensated by lower administrative costs at the macro- or micro-levels. Note that, however, provider level administrative costs are not measured routinely in established accounting frameworks such as the Organisation for Economic Co-operation and Development (OECD) System of Health Accounts. We thus are not able to make such comparison for a substantial number of countries.

 At the micro-level, the burden of registrations and administrative tasks for professionals is not captured in routine cost accounting data either, and often omitted by scholars of administrative costs in healthcare.^8^ However, in the Netherlands, general surveys continuously show high administrative burdens that circle around two days a week for professionals in hospitals and other providers.^9^ These surveys may measure perceived burdens more than actual time spent on administrative tasks. However, a new innovative measurement of actual time spend by Dutch general practitioner’s also finds that their administrative burden almost equals 40% and has increased over the past five years.^10^ To conclude, the total sum of all macro-, meso-, and micro-level related indirect costs might actually be around half of all healthcare spending in the Netherlands. Precise information is lacking and often not registered.

 That is why the fact that Zegers et al study a substantial amount of the total indirect and administrative costs – the burden of quality registries for professionals – into more depth is timely. However, such quality registries form also part of a broader data ecosystem where many interdependencies and connections do apply. We argue that a ‘solution’ to unnecessary high burdens of professional time for quality registries cannot be solved without adequate governance of all data and information systems, used within the wider healthcare system.

###  Thoughts About a Broader Governance of Data and Information for Healthcare

 Francis Lau and colleagues have developed a value framework for a coordinated strategy for the clinical adoption of electronic health records in Canada. They focus on the interconnectedness between the micro-, meso-, and macro levels and among other things point to the importance of governance issues, such as alignment with other reforms, incentive structures and developing interoperable technical infrastructures and national standards.^11^

 Good data governance is necessary to use such a value framework in policy practice. However, data governance in the Netherlands is complex. For one thing, the Dutch clinical registry landscape is quite scattered and un-coordinated as compared with the clinal registry landscape in countries like Sweden and Denmark. Dutch healthcare is governed through three different steering mechanisms – curative care (competition), long-term care (single purchasing), and social care (devolved to municipalities) – that need to co-produce and co-operate among the needs of complex patients with co-morbidities. The number of data custodians is among the highest in OECD countries.^12^ Some data custodians hold complex and even antagonistic relationships, for example insurance companies and hospitals each rely on their own data companies. Save prohibitions by privacy legislations, the oversight agencies can ask more or less all quality information from the providers they deem necessary for fulfilling their tasks, as can insurance companies (purchasers). However, data principals often do not share data with each other which adds to the burden of providers and professionals that need to provide the same data over and over. To sum up. This complex combination of competition that stipulates data as confidential, the private provision of care and insurance without a public clearinghouse, the necessary compliance to privacy regulations which create lots of confusion and hampers sharing of data all contributes to a lack of transparency and thus more request for data by individual stakeholders. On top, providers create their own databases and registries for specific purposes or they may choose to comply to registries of professional societies or accreditation bodies. A governance structure that strongly focuses on easy access to and sharing of reliable data is currently lacking.

 OECD has over the past years produced a series of international reports that demonstrate the heterogeneity between countries in the active use of data-linkage and optimizing the use of electronic health records, whilst assuring data-privacy and data-security. In 2017, OECD provided an official council recommendation on health data governance.^13^ Further implementation of these health data-governance recommendations and related optimization of data linkage practices and secondary data use of electronic health records might help pave the way to lowering the presently experience administrative burden on macro, meso and micro level of the Dutch healthcare system.

 Changing any system with an extensive legacy is difficult, but we do think a holistic approach that addresses both the issues of lack of adequate governance and operational efficiency in Dutch healthcare is necessary. The rigorous study by Marieke Zegers et al provides a strong factual basis for Dutch stakeholders to acknowledge the issues at hand. And with the coronavirus disease 2019 (COVID-19) crisis opening up many windows of opportunity for positive reforms,^14^ now may be the time to address this important concern in a fundamental way.

## Ethical issues

 Not applicable.

## Competing interests

 Authors declare that they have no competing interests.

## Authors’ contributions

 PPJ designed the commentary and wrote a first draft. Both LH and NK were responsible for substantial improvements in the manuscript.

## Authors’ affiliations

^1^Radboud Institute of Health Sciences (RIHS), Nijmegen, The Netherlands. ^2^Amsterdam Medical Center, Amsterdam, The Netherlands.
